# The Role of MALDI-TOF Mass Spectrometry in Photodynamic Therapy: From Photosensitizer Design to Clinical Applications

**DOI:** 10.3390/cimb47100834

**Published:** 2025-10-10

**Authors:** Dorota Bartusik-Aebisher, Kacper Rogóż, David Aebisher

**Affiliations:** 1 Department of Biochemistry and General Chemistry, Medical College, The Rzeszów University, 35-310 Rzeszów, Poland; dbartusikaebisher@ur.edu.pl; 2 English Division Science Club, Medical College, The Rzeszów University, 35-310 Rzeszów, Poland; kr117626@stud.ur.edu.pl; 3 Department of Photomedicine and Physical Chemistry, Medical College, The Rzeszów University, 35-310 Rzeszów, Poland

**Keywords:** photodynamic therapy, PDT, MALDI-TOF MS, photosensitizers, imaging mass spectrometry

## Abstract

Photodynamic therapy (PDT) has evolved considerably over the past decades, progressing from first-generation porphyrins to second- and third-generation photosensitizers, including nanocarrier-based systems with improved selectivity and bioavailability. In parallel, matrix-assisted laser desorption/ionisation time-of-flight mass spectrometry (MALDI-TOF MS) has become a gold standard for the characterisation of complex biomolecules, enabling precise determination of molecular mass, purity and stability. This narrative review explores the intersection of these two fields, focusing on how MALDI-TOF MS supports the development, characterisation and clinical application of photosensitizers used in PDT. Literature searches were performed across PubMed, Web of Science, Scopus and Base-search, followed by targeted retrieval of studies on MALDI and PDT applications. Findings indicate that MALDI-TOF MS plays a crucial role at multiple stages: confirming the synthesis and chemical integrity of novel photosensitizers, monitoring their metabolic stability in biological systems and characterising photodegradation products after PDT. Moreover, MALDI imaging mass spectrometry (MALDI-IMS) enables spatial mapping of photosensitizer distribution in tissues, while rapid pathogen identification by MALDI-TOF supports antimicrobial PDT applications. Collectively, the evidence highlights that MALDI-MS is not only a tool for molecular characterisation but also a versatile analytical platform with a direct translational impact on PDT. Its integration with other omics and multimodal imaging approaches is expected to enhance the personalization and clinical effectiveness of photodynamic therapy.

## 1. Introduction

### 1.1. Photodynamic Therapy (PDT)

#### 1.1.1. Principles of PDT

**Figure 1:**
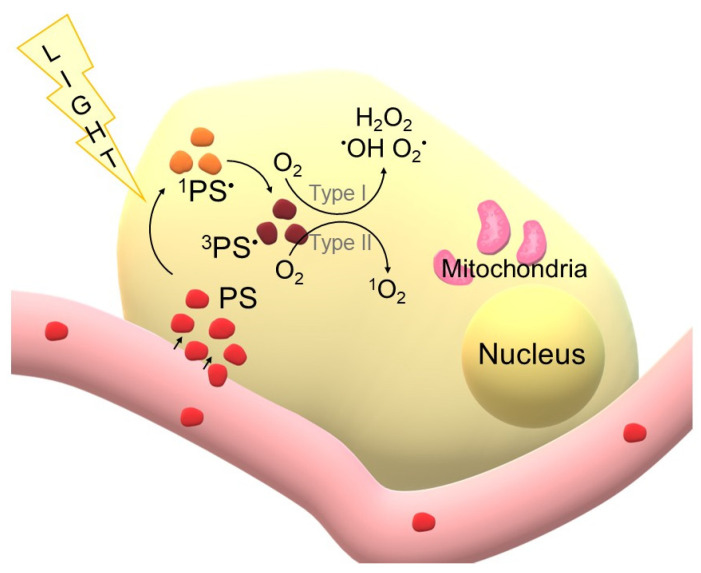
Schematic representation of photodynamic therapy (PDT) at the cellular level. Photosensitisers (PS) delivered via the bloodstream penetrate into the tumour cell. Upon light irradiation, PS molecules (red colour) are excited to the singlet state (^1^PS*) light braun colour) and then undergo intersystem crossing to the triplet state (^3^PS* (dark braun colour). In the presence of oxygen, two types of photochemical reactions occur: Type I reactions, leading to the formation of hydroxyl radicals (·OH), superoxide anions (O_2_·^−^) and hydrogen peroxide (H_2_O_2_), and Type II reactions, resulting in the production of singlet oxygen (^1^O_2_) (own elaboration).

Photodynamic Therapy (PDT) is a modern, minimally invasive treatment method that combines three essential elements: a photosensitiser (PS), light of an appropriately selected wavelength and molecular oxygen present in the tissues. When administered to the patient, the photosensitiser tends to selectively accumulate in lesional tissues such as tumours, pre-cancerous lesions or infectious foci. Illumination of these areas with light activates PS molecules, which go into an excited state and transfer energy to molecular oxygen. This results in the formation of reactive oxygen species (ROS), most notably singlet oxygen (^1^O_2_), responsible for the induction of damage to cell membranes, intracellular organelles and DNA. The cumulative effects lead to pathological cell death and, in addition, PDT can induce damage to tumour vasculature and modulate the host immune response. Thanks to its multidirectional activity and relatively high selectivity, photodynamic therapy is used both in oncology and in the treatment of certain dermatological or infectious diseases [1,2,3,4]. To better illustrate the molecular mechanisms underlying photodynamic therapy, a schematic representation of photosensitiser activation and subsequent ROS generation is shown in Figure 1.

The roots of photodynamic therapy date back to antiquity, when civilisations such as the Egyptian, Greek, Roman, Chinese and Indian used heliotherapy—treatment with sunlight and plant compounds (e.g., amiodin from *Ammi majus*). This is reflected in ancient texts such as the Ebers Papyrus dated 1550 BC. The term ‘heliotherapy’ was first used by Hippocrates in the 2nd century BC, when he taught about restoring health with the help of sun exposure [5]. In 1898, Oscar Raab, a student of Hermann von Tappeiner, discovered by accident that the combination of the dye acridine and light led to the death of Paramecium, while neither agent separately had a cytotoxic effect. This phenomenon laid the foundations for PDT [5,6]. The breakthrough came at the turn of the 20th century, when Danish physician Niels Finsen won the Nobel Prize (1903) for treating diseases such as lupus vulgaris with light rays. This was the first modern light therapy [7]. At the same time, in 1903, von Tappeiner, together with the dermatologist Jasionek, carried out the first clinical treatment of skin tumours (basal cell carcinoma) using a combination of dye (eosin) and light. The result was the complete cure of four out of six patients. In 1904, von Tappeiner coined the term “photodynamic” while pointing out that the presence of oxygen is essential for the biological effect [5,8,9,10].

Another breakthrough was the identification of the specific accumulation of porphyrins in tumours. In 1924, Policard observed the fluorescence of tumour tissues after exposure to porphyrins, and in 1948 Figge et al. confirmed the selective accumulation of porphyrins in tumour cells in animals [5,11]. Subsequently, in the 1960s, Lipson et al. developed the ‘haematoporphyrin derivative’ (HPD), which was used for diagnosis (tumour fluorescence) and PDT [12]. The real clinical breakthrough came when Thomas Dougherty and his Roswell Park Cancer Institute team conducted a PDT study using HPD to treat skin cancer (1978) [11,13]. The effectiveness of this therapy led to the coining of the term “Photodynamic Therapy” and the founding of the International Photodynamic Association (IPA) in 1986 [14]. In 1993, Photofrin^®^ (a commercial HPD) was approved as the first PS for the treatment of bladder cancer in Canada, which initiated its global clinical use [15]. In the following decade, the second and third generation of photosensitisers were developed. An example is verteporfin (Visudyne), approved by the FDA in 2000 for the treatment of the exudative form of AMD (macular degeneration)—the first PDT application outside oncology [16,17]. Since then, the development of PDT has included increasingly advanced PS (chlorines, phthalocyanines), better light sources, targeted methods, combination techniques (e.g., immuno-PDT, SPDT) and applications in a wide range of medical disciplines [14,18].

#### 1.1.2. PDT Components

**Figure 2:**
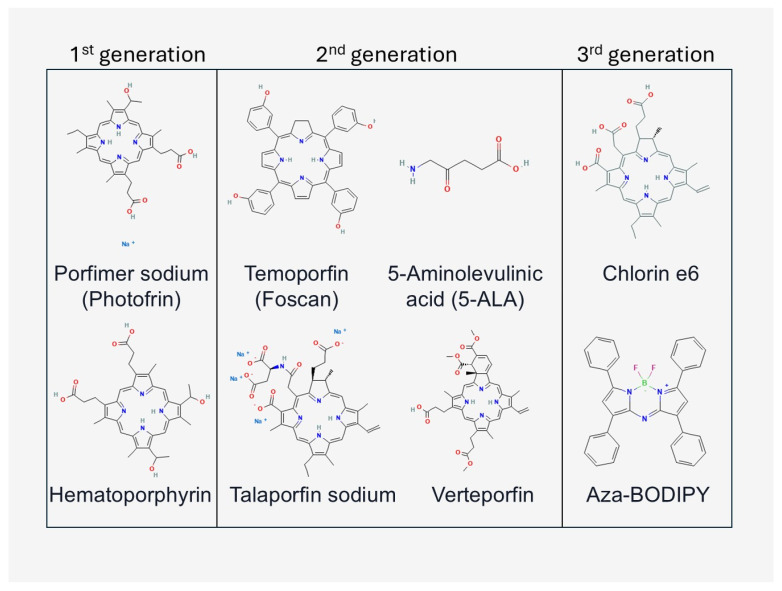
Examples of photosensitisers used in photodynamic therapy (PDT) by generation. Generation I includes classical porphyrin derivatives such as sodium porphimer (Pho-tofrin^®^) and haematoporphyrin. Generation II is represented by compounds with improved photophysical and pharmacokinetic properties, including temoporphine (Fos-can^®^), 5-aminolevulinic acid (5-ALA), talaporphine sodium (NPe6) and verteporphine (Visudyne^®^). Generation III includes modified and novel photosensitisers, e.g., chlorin e6 and aza-BODIPY derivatives, characterised by high selectivity and the ability to conjugate with nanocarriers. Formulations are from the PubChem database (NCBI, accessed 6 September 2025) (own elaboration).

A key element of photodynamic therapy is photosensitisers (PS), which are chemical compounds capable of being excited under the influence of light and generating reactive oxygen species [19]. The oldest and most studied group are the porphyrins and their derivatives, of which the first widely used clinical preparation was hematoporphyrin derivate (HPD) and its commercial form Photofrin^®^, approved for the treatment of bladder and oesophageal cancers [20,21]. In subsequent years, the so-called second generation of photosensitisers was developed, including chlorines (e.g., temoporphine—Foscan^®^) and phthalocyanines, characterised by stronger absorption in the red range and greater tissue selectivity. The third generation, on the other hand, consists of carrier-conjugated PS (nanoparticles, antibodies), which increase the specificity of accumulation in tumour tissues and reduce side effects [22,23,24,25]. We have shown a selection of frequently used photosensitisers in research and the clinic in Figure 2.

The correct choice of light source is crucial to the effectiveness of PDT, as only radiation with a wavelength corresponding to the maximum absorption of the photosensitiser is able to excite its molecules and initiate the formation of reactive oxygen species. Moreover, the choice of wavelength also determines the depth of penetration in the tissues and thus determines whether the therapy will be effective for superficial lesions or deeper tumours. Advances in light sources have been one of the key factors in the development of photodynamic therapy (PDT) [26]. The first clinical treatments were based on the use of broad-spectrum lamps (quartz, halogen, xenon or tungsten), which emitted radiation over a wide range of the electromagnetic spectrum. They had the advantage of simplicity of construction and low cost, as well as the ability to cover the absorption areas of various photosensitisers. However, they required the use of optical filters to reduce unnecessary wavelengths, which entailed the loss of some energy [27,28]. These lamps are mainly used today in dermatology for the treatment of superficial skin lesions [29].

A breakthrough was the introduction of lasers, which, thanks to their monochromaticity and coherence, allowed precise adjustment of the wavelength to maximise photosensitiser absorption. Among the first systems used were argon lasers (488–514 nm), krypton lasers and dye lasers, allowing infinite wavelength adjustment. In the following years, Nd:YAG lasers were also used, in which emission corresponding to porphyrin characteristics was achieved by frequency conversion. These lasers provided high efficacy and dose control, but their large size, high cost and limited mobility hindered their widespread clinical implementation [30,31,32]. The biggest revolution in practical applications has been brought about by diode lasers, which are now the standard in clinical PDT. These devices are compact, economical to operate and allow emission in the red light range (630–700 nm), corresponding to the therapeutic window of superficial tissues, as well as near-infrared wavelengths (up to 1000 nm), which penetrate deeper tissues. This allows the light to penetrate deeper layers to effectively treat mucosal, respiratory or urinary lesions. Diode lasers, due to their ease of use and high efficiency, are currently the most commonly used light source in clinical practice [33,34,35].

In parallel, light-emitting diodes (LEDs) are being developed and are widely used in dermatology, dentistry and antimicrobial PDT (aPDT). Compared to lasers, they have lower power and lack coherence, which limits their use for superficial lesions, but LEDs have the advantage of low cost, safety and the ability to cover large treatment fields. This makes them ideal for the treatment of acne, actinic keratosis, or biofilm infections [36,37,38,39].

Fibre-optic and endoscopic systems are an important complement, allowing light to be precisely delivered to hard-to-reach locations, including within the oesophagus, bladder, or bronchi. Fibre optics terminated with diffusers allow even illumination of larger tissue volumes, increasing the effectiveness of treatment of internally located tumours. Thanks to these developments, PDT is increasingly used in clinical oncology [40,41,42].

In recent years, the concept of so-called daylight PDT, using natural sunlight, has also emerged. This method is particularly valuable in the treatment of solar keratosis and skin lesions, as it reduces pain and simplifies the procedure, and part of the treatment can be carried out outside the clinical office [43,44].

The development of light sources has enabled a significant expansion of clinical applications of photodynamic therapy. The choice of a specific solution depends on the type of photosensitiser, the location of the lesion and the clinical conditions, and the variety of available devices makes PDT a method that is extremely flexible and adaptable to different fields of medicine.

#### 1.1.3. Models Undergoing PDT

Evaluating the efficacy of photodynamic therapy requires research models that vary in biological complexity, from simple cell cultures to clinical materials. Each allows different aspects of the therapy to be analysed: molecular mechanisms, pharmacokinetics of photosensitisers, immune response or clinical efficacy [45].

Cancer cell lines (e.g., HeLa—cervical cancer, A549—lung cancer, MCF-7—breast cancer, U87—glioma) and normal lines (e.g., fibroblasts, keratinocytes) are the primary tools in PDT research as a control for selectivity [25,46,47,48]. Two-dimensional cultures allow rapid testing of phototoxicity, analysis of molecular mechanisms (apoptosis, necrosis, autophagy) or study of ROS generation. However, their limitation is their lack of complex architecture and gradients of oxygen, nutrients and photosensitiser.

Three-dimensional models are becoming increasingly important. Cell spheroids allow the study of light penetration and PS and the differences in the responses of cells located in the tumour core compared to the tumour periphery. Organoids derived from the patient’s tissues provide an even better representation of the tumour microenvironment and heterogeneity, allowing testing of therapies under in vivo-like conditions, and providing a tool for personalising PDT [49,50].

The next step in translating the results is through animal models, mainly mice and rats, using xenografts (e.g., melanoma B16, carcinoma SCCVII, human lines in immunodeficient mice) or chemically induced tumours. These models make it possible to study not only the cytotoxic effect, but also the effect of PDT on angiogenesis, the tumour vascular system and the induction of the immune response. An important application is also the evaluation of the pharmacokinetics and pharmacodynamics of new photosensitisers, as well as the efficacy of light delivery systems (e.g., endoscopic probes, fibre optics) [51,52]. More advanced studies use transgenic models or spontaneous tumours in animals (e.g., dogs, cats), which better replicate clinical treatment conditions and can bridge the gap between laboratory studies and clinical trials [53,54].

Biopsy and resection tissues from patients are also an important tool in translational research. They allow the evaluation of photosensitiser accumulation, light distribution and PDT effects under conditions that correspond to the natural heterogeneity of the tumour, the presence of stroma and blood vessels. Molecular analyses of ex vivo material allow the identification of susceptibility and resistance markers, which supports the development of personalised medicine [55,56,57,58].

#### 1.1.4. Latest Developments in PDT

Recent years have seen several innovative applications of photodynamic therapy that significantly expand its clinical use. For example, vascular-targeted PDT with padeliporphin (TOOKAD^®^/padeliporphin VTP) has gained EMA approval for patients with locally limited, low-risk prostate cancer, offering an organ-saving alternative to radical treatment. A pivotal randomised trial demonstrated significant ‘cleaning’ of tumour foci and the potential to defer/avoid radical surgery in a significant proportion of patients; the EMA decision was based on data including 413 patients. This is the first widely accepted clinical implementation of vascular-targeted PDT in organ oncology, indicating the maturity of interstitial PDT technology and its potential for applications in other solid tumours available to interventional fibroids [59,60]. Recent studies and experimental clinical work show that PDT induces immunogenic cell death (ICD) and transforms the tumour microenvironment (e.g., vascular normalisation, lymphocyte influx), which sensitises it to checkpoint inhibitors. PDT + anti-PD-1/PD-L1 combinations show enhanced control of primary tumours and metastasis and are a harbinger of ablative effects; this is a direction with high translational potential for early phase studies in immunologically ‘cold’ tumours [61,62]. Nanocarriers carrying photosensitisers are also increasingly important. Nanoparticles—including liposomes, polymer micelles, dendrimers or inorganic nanoparticles (e.g., gold, silica, upconversion nanoparticles)—allow us to increase the bioavailability of PS, improve selective accumulation in the tumour (EPR effect) and protect the photosensitiser from degradation in circulation. Due to the possibility of surface modification, nanoparticles can be conjugated with receptor ligands or antibodies, increasing the specificity of the therapy [63,64]. Moreover, nanocarriers often integrate additional functions—e.g., oxygen delivery (perfluorocarbon), synergistic release of cytotoxic drugs or fluorescence and photothermal imaging capabilities—in a single system. Preclinical studies and early clinical trials are currently underway on PDT nanosystems, which have the potential to overcome key limitations of the method, such as tumour hypoxia and limited light penetration [65,66,67]. It is also important to develop an appropriate therapy model to overcome unwanted side effects such as pain during irradiation. New pain-free regimens—including daylight-mimicking and two-step PDT—significantly reduce pain while maintaining efficacy, which increases the possibility of scaling up therapy in daily practice (office, outpatient care). This is an example of advances not only in technology, but also in process, which translate into method adoption by the healthcare system [68,69]. One of the key challenges in photodynamic therapy is to precisely track the biodistribution of a photosensitiser in tissues and to identify the molecular effects of its action. Classical methods, such as fluorescence spectroscopy or histochemistry, provide limited spatial and quantitative information. MALDI-MSI (Matrix-Assisted Laser Desorption/Ionisation Mass Spectrometry Imaging) overcomes these barriers by allowing the visualisation of photosensitiser distribution and products of photodynamic reactions without the need for labelling [70]. This allows pharmacokinetic phenomena (absorption, accumulation, elimination) to be analysed with high spatial resolution and PDT-induced metabolic changes in the tumour microenvironment to be monitored [71,72,73]. MALDI is therefore becoming a key tool for understanding the mechanisms of therapy and optimising its clinical efficacy.

### 1.2. Matrix-Assisted Laser Desorption/Ionisation (MALDI)

#### 1.2.1. Principles of MALDI

The Matrix-Assisted Laser Desorption/Ionisation (MALDI) method is one of the soft ionisation techniques that have revolutionised mass spectrometry by enabling the analysis of large and fragile biomolecules without degradation [74]. The sample is mixed with a suitable organic matrix (usually aromatic acids, e.g., sinapic acid, α-cyanohydroxycinnamic acid) and then crystallised on a metal plate. A short laser pulse (usually UV) causes energy absorption by the matrix, which leads to rapid evaporation and simultaneous ionisation of the analyte. The key element is the charge transfer from the matrix to the analyte molecule, which allows low fragmentation molecular ions to be obtained [75,76]. MALDI-TOF (Time-of-Flight) combines this ionisation method with a time-of-flight analyser. After ions are produced in the MALDI source, they are accelerated in an electrostatic field and fall into a so-called TOF tube. Since all ions receive the same charge and kinetic energy, their speed (and thus their time to reach the detector) depends solely on the mass-to-charge ratio (*m*/*z*). Lighter ions arrive faster and heavier ions slower, allowing them to be separated and their masses to be accurately determined. The technique is characterised by very high sensitivity, a wide mass range and a high throughput of analysis [77,78].

Its origins can be traced back to the mid-1980s, when Franz Hillenkamp and Michael Karas described the organic matrix-assisted laser desorption process, in which the matrix absorbs the energy of laser radiation and transfers it to the analyte molecules, allowing them to ionise under mild conditions [79]. Independently of them, Koichi Tanaka developed an alternative ‘soft laser desorption’ method in 1987, in which he used cobalt microspheres suspended in glycerol as the absorbing medium. In this way, he was able to obtain mass spectra for large proteins, reaching above 100 kDa, for the first time. Although Tanaka’s technique differed from Karas and Hillenkamp’s classic MALDI, his work was recognised as a fundamental step in the history of soft ionisation development, earning him the Nobel Prize in Chemistry in 2002, along with John Fenn, the creator of ESI—electrospray ionisation [80,81]. The 1990s saw the rapid development of the MALDI-TOF method, a combination of MALDI and ion time-of-flight analysis. With this configuration, the possibility of fast, highly sensitive analysis of high-molecular-weight biopolymers was achieved [82]. MALDI-TOF has revolutionised biological research, enabling not only the identification of peptides and proteins, but also the characterisation of oligosaccharides, lipids and nucleic acids [78,83,84,85]. Particularly importantly, the method has a high resistance to the presence of contaminants such as salts or detergents, which makes it extremely useful in the analysis of biological samples [78]. Since the 2000s, MALDI-TOF has started to be widely used not only in basic research, but also in clinical and industrial diagnostics. A breakthrough application was in the identification of microorganisms, which has become standard in many microbiology laboratories since around 2010 [86,87]. Meanwhile, MALDI imaging (MALDI-IMS), the imaging of the distribution of biomolecules in tissue sections, has been developed, with applications in oncology, neuroscience and pharmacology [88,89,90]. Another important step was the introduction of tandem techniques, such as MALDI-TOF/TOF or MALDI-qTOF, enabling more accurate structural analysis, including the study of peptide sequences and characterisation of post-translational modifications of proteins [91,92,93]. MALDI is currently used extensively in proteomics, metabolomics, polymer, drug and nanomaterial research, as well as in microbial diagnostics and environmental analysis [94,95].

#### 1.2.2. MALDI System Components and Research Materials

The MALDI system consists of several key components that work together to enable efficient desorption, ionisation and analysis of high mass molecules. The key components include a laser source, matrix, sample application plate, ionisation and vacuum chamber, mass analyser and detector. Each of these plays a specific role in the measurement process, but it is the matrix that is the critical element, determining the range of analyses possible and the quality of the spectra obtained [96].

The most commonly used radiation sources in MALDI are pulsed lasers emitting in the UV range, such as the nitrogen laser (N_2_, 337 nm) or Nd:YAG with third harmonics (355 nm). Their parameters—wavelength, pulse energy and repetition rate—determine the efficiency of energy absorption by the matrix and the degree of ionisation of the analyte [97,98,99]. Some variants also use IR lasers, but their use is limited to specific applications, e.g., analysis of highly infrared-absorbing molecules [98].

**Figure 3:**
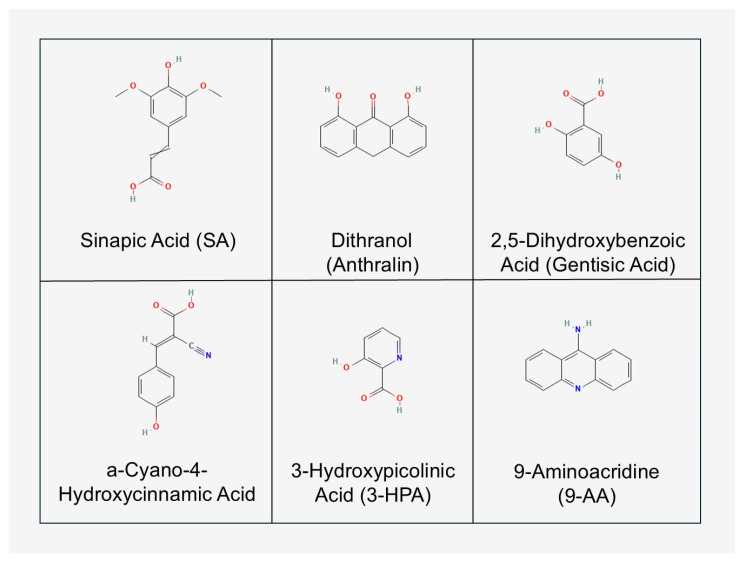
Examples of common matrices used in the MALDI technique. Organic matrices include: Sinapic acid (Sinapic Acid, SA), dithranol (Anthralin), 2,5-dihydroxybenzoic acid (Gentisic Acid, 2,5-DHBA), α-Cyano-4-Hydroxycinnamic Acid (CHCA), 3-HydroxyPicolinic Acid (3-HPA) and 9-Aminoacridine (9-AA). The choice of matrix depends on the type of analyte to be analysed and the type of ions to be generated. Formulas are from the PubChem database (NCBI, accessed 6 September 2025) (own elaboration).

A matrix is a low-molecular-weight organic compound whose role is to absorb laser energy and transfer it to the analyte molecules under mild conditions, allowing ionisation without significant fragmentation. The selection of a suitable matrix is crucial, as it affects both the sensitivity and specificity of the method [100]. The most commonly used matrices include the following: (1) For peptides and proteins: sinapic acid (SA) and α-cyanohydroxycinnamic acid (CHCA) [101,102]; (2) For oligonucleotides and large biomolecules: 3-hydroxypicolinic acid (3-HPA) [103]; (3) For lipids and small-molecule metabolites: 2,5-dihydroxybenzoate (DHB), norharmane or 9-aminoacridine (9-AA), often used in negative ionisation mode [104]; (4) Modern alternative matrices: metal nanoparticles (Au, Ag, TiO_2_), graphene and carbon materials that reduce background interference and enable so-called “matrix-free MALDI” [105,106,107,108]. Criteria for matrix selection include the following: (i) spectral compatibility with the laser wavelength, (ii) ability to crystallise in the presence of the analyte, (iii) low background signals in the mass range of interest, (iv) stability during imaging analysis [109,110,111,112]. Figure 3 shows examples of the matrices used during MALDI.

**Figure 4:**
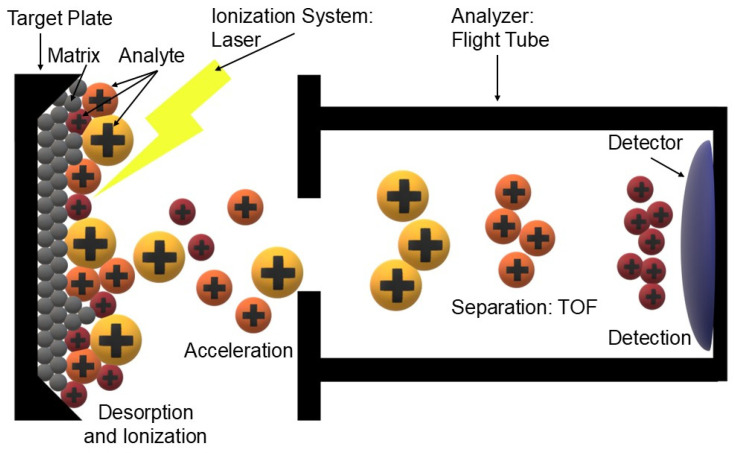
Schematic representation of the MALDI-TOF spectrometer. The sample, consisting of analyte and matrix, is placed on a plate (Target Plate). A laser pulse causes desorption and ionisation of the molecules (desorption and ionisation). The generated (+ positive ions) ions are accelerated in an electrostatic field (acceleration) and then separated in a time-of-flight tube (separation: TOF) depending on the mass-to-charge ratio (*m*/*z*). The final registration of the signal takes place in the detector (detection) (own elaboration).

Samples are applied to plates (so-called target plates), usually made of stainless steel or coated with special coatings that improve matrix crystallisation. The uniform distribution of the crystals has a direct impact on the reproducibility of the spectra and the sensitivity of the analysis. The MALDI process takes place under high vacuum conditions, which minimises ion collisions and increases resolution. The ion source contains electrodes and electrostatic lenses that direct the resulting ions to the mass analyser. In newer systems, atmospheric variants (AP-MALDI) have been developed, allowing samples to be analysed directly under near-natural conditions. The most commonly used analyser in MALDI is TOF (Time-of-Flight), allowing the detection of a wide range of masses with high sensitivity. For more advanced analyses, TOF/TOF (ion fragmentation), Orbitrap or FT-ICR systems are used, providing the highest resolution and measurement accuracy. Ion detection is usually carried out using microchannel particle (MCP) systems or hybrid detectors, which record both the intensity and time of ion arrival [113,114,115]. We have included a schematic representation of MALDI-TOF in Figure 4.

The MALDI technique allows the analysis of an extremely broad spectrum of materials—from pure biomolecules to complex tissues or clinical samples. One of the classical and most common applications of MALDI-TOF is proteomic analysis: the identification and characterisation of proteins and peptides, allowing the determination of molecular masses, sequences and post-translational modifications. Short protein fragments (peptides) are also analysed in further structural analysis by tandem MALDI MS/MS methods [116]. MALDI is also used in the analysis of small DNA fragments, oligonucleotides and genetic analyses (e.g., SNP genotyping or mutations), often in combination with PCR techniques and specialised matrices [78]. The MALDI technique, especially in the imaging mode (MALDI-IMS), allows spatial mapping of lipids, metabolites and other small molecules in tissues. This makes it possible to analyse the distribution of metabolic substances or biomarkers in a spatial context, e.g., in tumours [117,118]. MALDI-IMS enables direct examination of thin tissue sections, allowing simultaneous analysis of multiple compounds without labelling. The technique has wide applications in oncology, pharmacology and neuroscience, including mapping drug distribution and biomarkers [117,119,120]. MALDI-TOF MS has revolutionised microbiological diagnostics—it enables rapid, precise identification of bacteria and fungi based on a ‘mass fingerprint’, which has become the standard in clinical laboratories [78,121]. The MALDI technique is also used in the analysis of synthetic materials—polymers and oligomers. It allows the molecular weight and mass distribution to be determined with a high degree of accuracy, which is important in materials-technology research [122]. MALDI represents a versatile analytical technology, capable of analysing biomolecules, metabolites, tissues and clinical and synthetic materials. Thanks to its versatility, it has found applications in research and clinical practice.

#### 1.2.3. Recent Applications of MALDI

The MALDI technique is developing rapidly, already entering beyond the classical framework of proteomics and microbial diagnostics. MALDI-TOF MS has revolutionised microbiological diagnostics, reducing the identification time of bacteria and fungi by up to 24 h compared to biochemical techniques, significantly improving the workflow in clinical laboratories [123]. Recent reports also point to the development and implementation of methods that directly detect antibiotic resistance mechanisms, including detection of β-lactamase enzymes and other resistance markers [124,125]. MALDI-TOF MS is increasingly used in food quality control to rapidly identify microorganisms, monitor contamination and trace the origin of microflora in food products. In this way, it can support outbreak prevention and consumer safety activities [126]. Advanced reactive matrices (e.g., fluoromethyl-pyridine) have made it possible to image neurotransmitter systems (dopamine, serotonin and their metabolites) with a spatial resolution of up to 10 µm, both in parkinsonian models and in human post-mortem tissues. This method significantly increases the sensitivity and precision of MALDI-MSI analysis in neurological studies [127]. Combining the MALDI technique with photodynamic therapy (PDT) is also becoming increasingly important.

### 1.3. Common Point of PDT and MALDI

**Figure 5:**
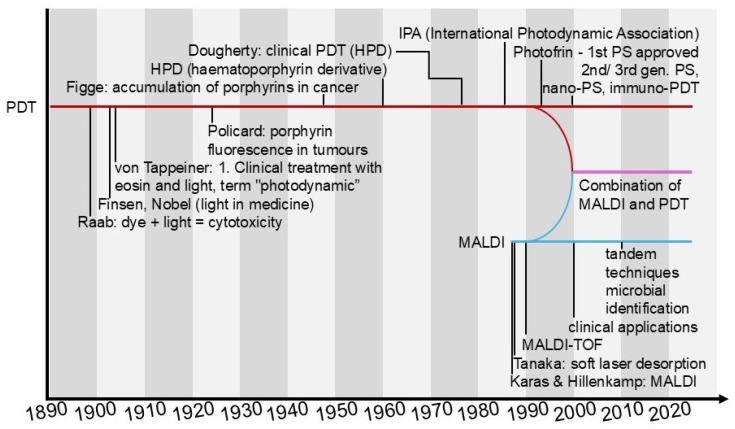
Timeline showing the parallel development of photodynamic therapy (PDT) and MALDI MS techniques. Initially, the two fields developed independently, but in recent years they have begun to intermingle, finding common applications in photosensitiser research and the therapy itself (own elaboration).(Braun colour main axis with PDT development, pink colour axis, development of PDT and MALDI combination, blue colour axis, development of MALDI).

With the development of photodynamic therapy (PDT), more and more advanced photosensitisers have been developed, ranging from classical porphyrins, chlorines and phthalocyanines to modern nanocarrier conjugates that provide better selectivity and bioavailability [2,128]. During the same period, MALDI-TOF-based mass spectrometry has established itself as the gold standard in the characterisation of complex biological molecules, enabling the precise determination of the mass, purity and stability of synthesised compounds [77]. It is only in recent years that the two fields have begun to closely intertwine: MALDI-TOF MS has become an important tool in the verification of the synthesis of new photosensitisers and in the study of their interactions with biomolecules. The first reports on the use of MALDI-TOF MS on photosensitisers date back to 1999, when Kasselouri et al. investigated the repetition of the photoproduct meta-tetra(hydroxyphenyl)chlorin (m-THPC), a second-generation PS [72]. The integration of these two technologies enables not only the rapid confirmation of the structure and purity of photosensitisers, but also the assessment of their stability under biological conditions, which is of direct relevance for the further development and optimisation of PDT [73,129,130,131]. To illustrate the key milestones in the development of both PDT and MALDI, as well as the timing of their integration, we have prepared a diagram in the form of parallel timelines. It depicts key historical events, technological breakthroughs and points of commonality between the two fields (Figure 5).

In this review, we have highlighted the MALDI-TOF MS technique as an important tool to support work on photosensitisers used in PDT. MALDI-TOF combines several features useful in the context of PS synthesis and validation: rapid and simple sample preparation, ‘soft’ ionisation allowing detection of intact mass bioconjugates (e.g., PS-antibody conjugates), relatively high tolerance to salts and impurities and the ability to spatially image (MALDI-IMS) the distribution of PS in tissues [116,117,118]. These properties make MALDI-TOF particularly useful for quality control, evaluation of conjugation stoichiometry, metabolite and photoproduct monitoring and biodistribution mapping—key tasks in the development and optimisation of PDT therapies. At the same time, the technique has limitations (e.g., problems with quantitative analysis of low-molecular-weight compounds at low *m*/*z*, or lower mass resolution compared to FT-ICR/Orbitrap), highlighting the need for its complementary use with other analytical methods [77,96,116].

## 2. Materials and Methods

This article is a narrative review of the literature on MALDI applications in photodynamic therapy (PDT). As a first step, a database search was conducted using the following phrase: ‘MALDI AND PDT’, which yielded a total of 426 results: 42 in PubMed, 101 in Scopus, 98 in Web of Science and 185 in Base-search. After removing duplicates, 49 unique publications were selected for further analysis.

In a second step, the search was expanded to include related terms such as “MALDI”, “PDT”, “photodynamic therapy history”, “MALDI history” and similar phrases, in order to capture reports not included in the first stage but relevant to MALDI applications in the context of PDT and the history of the development of the MALDI technique and PDT.

**Figure 6:**
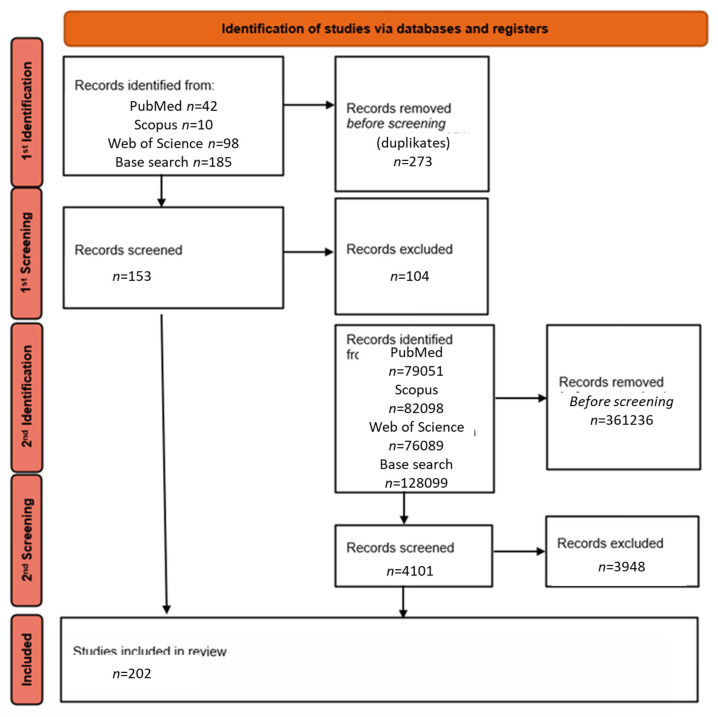
Literature selection process (PRISMA-style adapted flowchart for narrative review). The letter “*n*” stands for the word “number” (own elaboration).

After applying the inclusion criteria, mainly original clinical and preclinical studies (prospective and retrospective), systematic reviews, meta-analyses and English-language publications from the last 5 years or so, excluding papers not directly related to the topic, were retrieved, and a total of 182 publications were finally selected for detailed analysis. The literature selection process is presented in Figure 6 in the form of a PRISMA-style literature flow diagram adapted to the narrative review methodology.

## 3. Photosensitizers Checked with MALDI

MALDI-TOF MS can be a useful tool while still at the stage of synthesising potential photosensitisers. Newly obtained phthalocyanines (triazole, coumarin, Schiff-like), BODIPY-Ir/Ru complexes and metallophthalocyanines (Zn, Si, Cu, Co, Mg and lanthanides: Lu, Sm, Y) are candidates for PDT. These compounds have shown favourable photophysical and photochemical properties, such as good solubility in organic solvents, adequate excited state time and the ability to generate singlet oxygen at levels acceptable for PDT applications. Their accurate molecular weight analysis using the MALDI-TOF MS technique allows full chemical characterisation. Although imaging in biological materials was not performed in this study, the fact that these compounds give clear signals in MALDI suggests the potential for their subsequent use in imaging photosensitiser distribution in tissues—provided the appropriate biological and analytical criteria are met [132,133,134,135,136,137,138,139,140,141,142,143,144,145,146,147,148,149,150]. The MALDI-TOF technique allows confirmation of the molecular weights and fragmentation patterns of newly synthesised photosensitisers prior to their use in photodynamic therapy. MALDI-TOF thus provides an element of quality control and chemical structure confirmation. However, it should be borne in mind that it provides limited information on topology and isomerism—in this respect, it is necessary to supplement NMR/HR-MS/X-ray [140,151]. Recently, there has been growing interest in the use of nanostructures in medicine, particularly as smart carriers of photosensitisers for photodynamic therapy (PDT). The MALDI-TOF technique makes it possible to monitor and confirm the construction stage of such hybrid nanomaterials, providing reliable control of their formation process [152,153,154]. MALDI-TOF MS also plays an important role in the chemical and structural control of complex therapeutic systems. An example is a recently published study of a novel ruthenium (II) complex, Ru-NH2, with potent near-infrared (NIR) phototoxic activity, which was additionally conjugated to the antibody cetuximab (CTX) to achieve selective delivery to cancer cells. In this study, MALDI-TOF mass spectrometry was used to confirm the efficiency of the bioconjugation process and accurately determine the number of ruthenium fragments attached to the antibody. The mass spectra obtained unambiguously showed the presence of up to four molecules of the complex attached to a single antibody molecule. Such analysis was crucial to validate the structure and stoichiometry of the conjugates prior to further biological studies [155]. The use of MALDI-TOF MS in this context demonstrates that this technique can be a tool to support the development of next-generation photodynamic therapy, enabling the precise characterisation of complex bioconjugates, the assessment of the degree of modification of macromolecules, as well as the quality of synthesised photosensitisers. Furthermore, the ability to distinguish fine mass differences makes MALDI-TOF an ideal technique for rapid analysis of biological and therapeutic samples in research and potentially clinical settings. In order to summarise and systematise the current state of knowledge on the use of MALDI spectrometry in photosensitisation (PS) studies, the most important examples are summarised in tabular form (Table 1). Both information on the class of compounds and their theoretical or determined mass in MALDI experiments, as well as the matrices used and the main conclusions of the analyses are included.

In general, the criteria for selecting photosensitizers for PDT include: (i) strong absorption in the therapeutic window (650–800 nm) and efficient singlet oxygen generation, (ii) chemical stability and suitability for formulation in drug delivery systems, (iii) preferential accumulation in target tissues with minimal dark toxicity, (iv) translational feasibility, such as biocompatibility and regulatory acceptance [11,19,20,21]. Figure 2 illustrates several representative structures of photosensitizers currently being investigated in PDT, including porphyrins, chlorins and phthalocyanines. These molecules were selected as examples to highlight the chemical diversity of compounds used in this field and are not intended to represent the full spectrum of available PS. From the perspective of MALDI-TOF MS, additional considerations include the ionisation efficiency of the PS and their compatibility with matrix preparation protocols, which affect detection sensitivity and reliability. Thus, while the compounds shown in Figure 2 serve as illustrative examples, a wide range of other structures—including second- and third-generation PS such as chlorins, aza-BODIPY dyes, or PS conjugated to nanoparticles and antibodies—can be employed in PDT and successfully characterised by MALDI-MS [22,23,24,25].

## 4. MALDI at the Administration of the Compound and Its Metabolism In Vivo During and After PDT

**Figure 7:**
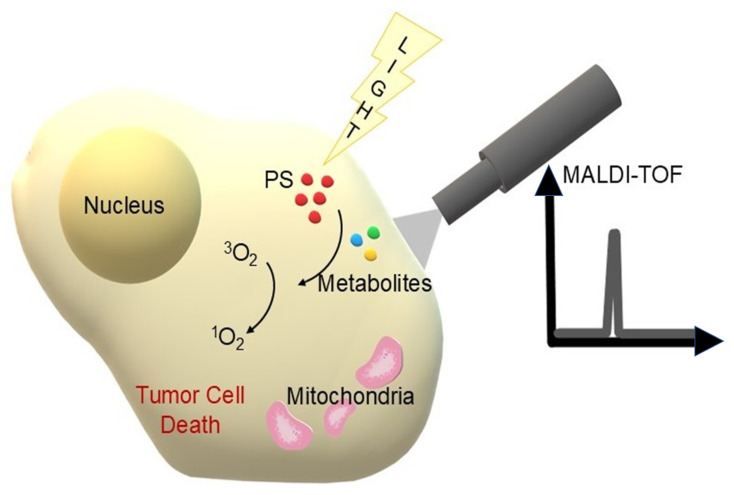
Schematic representation of photosensitiser (PS) metabolism following PDT activation and reactive oxygen species (ROS) generation. Degradation and transformation of PS leads to the formation of various metabolites, which can then be identified and analysed by MALDI-TOF MS (own elaboration).

While MALDI-TOF MS has traditionally been applied to the structural confirmation and quality control of photosensitizers, its translational potential is increasingly recognised in biological and clinical contexts. Recent studies demonstrate that MALDI can be employed to monitor photosensitizer metabolism in cancer cells, evaluate their stability in vivo and characterise treatment-induced proteomic changes, thereby bridging the gap between molecular design and therapeutic outcome. Moreover, the integration of MALDI-based imaging with functional assays provides direct insight into photosensitizer biodistribution within tumours and surrounding tissues, supporting more accurate predictions of therapeutic efficacy. These developments underscore the clinical relevance of MALDI-TOF MS as a versatile platform extending beyond chemical validation to real-world biomedical applications. In a study on tri(glucosyloxyphenyl)chlorin (TPC(m-O-Glu)3), one of the promising glycoconjugated photosensitisers, MALDI-TOF MS allowed detailed monitoring of the gradual deglycosylation of this compound in HT29 cancer cells. Mass spectra showed the appearance of signals corresponding to products of the loss of further glucose units (*m*/*z* 989, 827, 665), as well as further products of the oxidation of chlorinated macrocycle to porphyrins (e.g., TPP(m-OH)3). These data, complemented by HPLC analysis, allowed the construction of a kinetic profile of PS metabolism over time. It was shown that the final metabolite—a completely sugar residue-free and oxidised porphyrin compound—had significantly lower photobiological activity compared to the starting compound [131]. MALDI-TOF can also be used to monitor the stability of photosensitisers used in photodynamic therapy. Laville et al. found, for example, that after incubation of glycoconjugated porphyrin molecules in retinoblastoma cells in vitro, only minor cleavage of the O-glycosidic bonds occurred, while the stability of the S-glycosidic analogues remained high. This makes it possible to select suitable photosensitisers for downstream studies and subsequent therapy [159]. The use of MALDI-TOF in this case, therefore, provides an example of directly combining analytical techniques with PDT efficacy assessment, highlighting the importance of chemical stability and resistance to cellular metabolism for the real activity of a photosensitiser in vitro and in vivo. In the study of combination therapies, combining photodynamic therapy with chemotherapy, it is crucial to simultaneously determine the distribution of the photosensitiser and cytotoxic drug within the tumour cells. Kannen et al. used MALDI-TOF with a zeolite matrix, enabling simultaneous detection of the photosensitiser protoporphyrin IX (PpIX) and docetaxel. This approach allowed direct measurement of the uptake of both compounds in PC-3 (sensitive) and PC-3-DR (docetaxel-resistant) cells while maintaining the morphological integrity of the cells. The results showed that while both PpIX and docetaxel were detected in PC-3 cells, only PpIX signal was present in PC-3-DR cells, confirming the selective loss of docetaxel uptake capacity of docetaxel-resistant cells [160]. The same researchers used imaging mass spectrometry (IMS) with laser-assisted ionisation (MALDI) to image an anti-cancer drug (docetaxel) in prostate cancer (PC-3) cell lines. To increase detection sensitivity, the MALDI matrix was optimised using a mixture of NaY5.6 zeolite and 6-aza-2-thiomine (ATT), which allowed a 13-fold increase in the signal of the sodium adduct ion [M + Na]+ of docetaxel compared to the conventional matrix. The photosensitiser, in this case protoporphyrin IX (PpIX), was imaged by fluorescence and then detected in the same samples using CHCA matrix IMS and a spray method. Combining the fluorescence images (PpIX) with the maps obtained by MALD-IMS (docetaxel) allowed simultaneous analysis of the distribution of both therapeutic agents in cells. Such a strategy allows the precise evaluation of the efficacy of combination therapies, including PDT, by monitoring the location and potential interaction between photosensitiser and chemotherapeutic agent within the tumour [161]. To illustrate the role of MALDI-TOF mass spectrometry in the analysis of photosensitising transformations, a diagram (Figure 7) was prepared to illustrate the link between PDT activation, metabolite formation and their detection by MALDI-MS.

MALDI-TOF MS was already used more than 25 years ago to analyse photodegradation products after PDT. The photosensitiser, meta-tetra(hydroxyphenyl)chlorine (m-THPC), was treated with 514 nm light to reproduce PDT conditions. The products were separated by HPLC and analysed using MALDI-TOF MS. Analysis revealed the formation of a number of hydroxy derivatives of m-THPC and products resulting from the disruption of the pyrrole ring [72]. In studies using Photofrin^®^-PDT and ALA-PDT, MALDI has shown both photosensitiser consumption and significant changes in tumour protein profiles, providing a basis for fine-tuning therapy parameters to the actual needs of the patient [162]. In contrast, in HT29 cells treated with Foscan^®^, the MALDI-TOF technique was used to detect the presence of singlet oxygen. Moreover, the combination of MALDI with proteomic analysis (2D SDS-PAGE + MALDI-FT-ICR/MS) allowed the identification of changes in the protein profile of PDT-treated cells, indicating, among other things, the role of proteins of the disulfidoisomerase family in the cellular response [163]. Using MALDI-TOF MS, the difference in tumour protein expression of lung cancer cells (A549) after PDT with the photosensitiser EDAHB (2-demethoxy-2,3-ethylenediamino hypocrellin B) was also analysed. The use of two-dimensional electrophoresis (2-DE) combined with MALDI-TOF MS protein identification enabled the detection of 15 significantly altered proteins in response to PDT. One of the key findings was a significant downregulation of PKM2 (pyruvate kinase type M2), an enzyme that regulates aerobic glycolysis characteristic of cancer cells (Warburg effect). Further functional analyses confirmed that a decrease in PKM2 levels is associated with decreased lactate production and increased cancer cell death. Overexpression of PKM2 reduced the cytotoxic effect of PDT, while its silencing (RNAi) enhanced the therapeutic effect [164]. The study by Halada et al. used a combination of 5-aminolevulinate-based PDT (ALA-PDT) and mass spectrometry techniques to identify proteins in treatment-affected HL60 cells. Again, 2-DE was used, which showed significant changes in the protein profile after ALA-PDT. The use of MALDI and microcapillary liquid chromatography coupled with tandem mass spectrometry allowed the identification of 12 proteins with altered intensity or localisation associated with endoplasmic reticulum, mitochondrial, ribosome and cytoplasmic function [165]. In another study, PDT was performed with a hexyl derivative of aminolevulinic acid (HAL). Using 2-DE, a significant shift in the isoelectric point was revealed, suggesting, among other things, phosphorylation, acetylation or oxidative modifications of cysteine residues. Subsequently, through the use of MALDI-TOF MS, a group of proteins with altered expression and carbonylation were identified, including cytoskeleton proteins (vimentin, β-actin), chaperone proteins (HSP60, HSP70, GRP94) and energy pathway enzymes. These results indicated that HAL-PDT triggers a complex cellular response, including cytoskeletal disruption, modulation of energy metabolism and activation of oxidative stress mechanisms [166]. In another study, a prostate cancer cell model was subjected to PDT with pheophorbide and the MALDI-TOF technique identified key proteins regulating GDP-GTP exchange in the Ras protein family. A decrease in translationally controlled tumour protein (TCTP) and an increase in GDP dissociation inhibitor (GDI) were observed, leading to the maintenance of Ras proteins in an inactive state. These results suggest that one important mechanism of the anti-tumour effect of PDT is the blocking of the signalling pathway responsible for cell proliferation [167]. High-performance liquid-chromatography-coupled mass spectrometry (LC-MALDI-MS), supported by specialised DeepQuanTR software, provides a tool to quantitatively analyse proteome changes induced by various therapeutic interventions, including PDT. By adopting a label-free approach and using in-house standards, it is possible to analyse large sample sets without the need for isotopic labelling. DeepQuanTR enables the analysis of up to 100 samples simultaneously, allowing the detection of differentially expressed proteins that can be biomarkers of PDT efficacy or indicators of the body’s response to treatment, such as inflammatory response, tumour cell necrosis or remodelling of the tumour microenvironment. A study by Fugmann et al., mainly on renal and liver tumours, demonstrated the efficacy of this method in detecting changes in extracellular matrix proteins (e.g., fibronectin, tenascin-C) [168]. Furthermore, studies using MALDI-TOF spectrometry have shown that mitochondrial cardiolipin (CL) peroxidation plays a key role in PDT with 5-ALA. Oxidised CL disrupts cytochrome c binding, leading to apoptosis of cancer cells. MALDI-TOF MS allows precise monitoring of these changes and confirms that the GPX4 enzyme can protect mitochondria from damage, limiting the effectiveness of PDT [169]. MALDI-IMS also enabled spatial analysis of the presence of betulinic acid (BA) in tumour tissue after PDT. Although BA showed pronounced phototoxic activity in vitro under LED illumination, mass spectrometry imaging did not reveal the presence of BA in analysed tumour sections after in vivo administration. Integration of PDT with the MALDI-IMS technique allowed researchers to not only assess the efficacy of the therapy, but also to identify potential limitations related to the bioavailability of the photosensitiser in the tumour environment [170].

## 5. MALDI-TOF Detects Infection and Allows Rapid Treatment of PDT

MALDI-TOF MS has emerged as a powerful diagnostic tool for the rapid detection of microbial infections, which is directly relevant to the clinical application of PDT in both oncology and antimicrobial therapy. The mechanism underlying pathogen identification by MALDI-TOF relies on the generation of protein “fingerprints,” primarily derived from abundant ribosomal proteins in the mass range of 2–20 kDa. Each microorganism exhibits a unique spectral profile that can be compared to extensive reference libraries, enabling accurate species-level identification within minutes [171,172]. In addition, recent developments allow the detection of resistance-associated proteins, further refining therapeutic decision-making [173,174]. This rapid diagnostic capability is of particular importance in the context of photodynamic therapy, as microbial infections frequently complicate malignant wounds, postoperative sites and immunocompromised patients. By quickly identifying the causative pathogen, MALDI-TOF MS facilitates the selection of appropriate photosensitizers with antimicrobial activity and supports the immediate initiation of antimicrobial PDT, especially in cases involving multidrug-resistant organisms [175,176]. Thus, the integration of MALDI-TOF MS into PDT workflows holds promise for accelerating diagnosis-to-treatment pathways and improving outcomes in both oncological and infectious disease settings.

In the case of Liu et al., and also Li et al., this technique allowed an identification result to be obtained within minutes of strain isolation, allowing PDT to be used in combination with appropriate antibiotic therapy in an infection in the left thigh caused by Mycobacterium fortuitum and in an abscess with the pathogen Mycolicibacterium mucogenicum in the right back [87,88]. Also, Xing et al. used MALDI-TOF MS to identify Mycobacterium abscessus strains isolated from skin infections, which allowed the use of PDT with 5-ALA as an adjunct to antibiotic therapy. This significantly improved the efficacy of the treatment and shortened the recovery time [177]. MALDI-TOF has played a key role as a tool for the precise identification of the bacterial microflora present in the root canal in chronic periapical periodontitis, as well as in implant infections [178,179].

By using MALDI-TOF MS, it was possible to unambiguously detect and classify bacterial strains before and after treatment—both classical and photodynamic. Complete elimination of microorganisms was recorded in more than half of the cases after PDT with phenothiazine chloride, as confirmed by both quantitative (CFU) testing and qualitative MALDI-TOF identification [178]. In the study by Burchard et al., antimicrobial photodynamic therapy (aPDT) was performed as a complementary method for the elimination of oral biofilms. The use of aPDT with VIS and wIRA light and the photosensitiser ICG allowed not only a significant reduction in the number of viable bacteria, but also a modification of the microbial composition of the biofilm. MALDI-TOF MS played a key role in assessing the efficacy of the treatment, enabling precise identification of the bacteria that survived the therapy [180]. The use of MALDI-TOF allows accurate analysis of the type and variability of bacterial strains in the biofilm after treatment. In the Al-Ahmad et al. study, a reduction in the number of strains from 20 to only 4–6 and a shift in dominant species (e.g., elimination of Fusobacterium nucleatum and a significant reduction in streptococci) were observed after PDT [181].

## 6. Future Perspectives

Modern MALDI MS techniques are opening up new perspectives in the context of the therapeutic application of PDT. MALDI-IMS allows direct, non-invasive analysis of the distribution of photosensitisers and their metabolites in tissue sections, while maintaining high spatial resolution and molecular specificity. This makes it possible to map the distribution of PS in the tumour and control their penetration into tissues [175,182]. In the future, MALDI-IMS may become the standard method for assessing PS biodistribution, supporting the optimisation of dose and retention time in PDT, which is crucial for treatment efficacy and safety [74,161]. This technique, with its ability to analyse multiple fragments simultaneously, can become the basis for personalised photodynamic intervention, responding to tumour heterogeneity and individual patient characteristics.

In parallel, there is a strong trend towards the integration of MALDI-MS with other analytical techniques in a multi-omics approach. Combining data from proteomics, metabolomics and lipidomics allows us to understand the complex effects of PDT on the tumour microenvironment, repair mechanisms, energy metabolism and tissue structures. Although MALDI-IMS can provide a wealth of information on its own, it relies more on a selective molecular ‘picture’; it is only when integrated with other omics that it provides full insight into biological processes [183,184].

A further step will be the development of multimodal molecular imaging, integrating MALDI-IMS with techniques such as immunochemical microscopy, vibrational spectroscopy (e.g., Raman) and even in vivo imaging (e.g., MRI). This approach will enable the combination of molecular and morphological information, facilitating the analysis of tumour growth, the identification of regions most sensitive to therapy and the monitoring of the response to PDT in real time. While MALDI-IMS provides high-resolution molecular maps of photosensitizer and metabolite distributions in tissue sections, its limitation is that it remains an ex vivo technique [185,186,187]. In contrast, MRI offers non-invasive, real-time monitoring of PS biodistribution and tumour physiology, including perfusion, oxygenation and structural changes, but with lower molecular specificity [188]. The combination of these approaches could therefore provide complementary information: MRI enabling longitudinal in vivo tracking of photosensitizers or nanoformulations, and MALDI-IMS validating and refining molecular details at the tissue level [185,186,187,188]. Such integration may guide optimisation of PS dose, light exposure time and treatment scheduling, ultimately enhancing both the safety and efficacy of PDT. In the clinical setting, multimodal strategies linking MALDI-based ex vivo analyses with MRI-based in vivo monitoring could pave the way for more accurate patient stratification, improved treatment planning and personalised photodynamic interventions [189].

All of the MALDI applications described in this thesis—both in the characterisation of photosensitisers, studies of their metabolism in vivo and after PDT, and in microbiological diagnostics related to photodynamic therapy—remain an area of intensively developing research. Their further refinement and integration with modern analytical techniques are essential to fully realise the potential of MALDI in the development of effective and personalised PDT therapies.

## 7. Limitations

Although MALDI-TOF MS provides a powerful and versatile analytical platform in the context of photodynamic therapy, its limitations must also be considered to provide a balanced perspective. One major challenge lies in the analysis of hydrophobic photosensitizers, such as porphyrins or phthalocyanines, which often exhibit low solubility in conventional solvents and poor ionisation efficiency under standard MALDI conditions. This frequently requires the use of non-standard matrices, co-crystallisation techniques, or chemical derivatization, all of which can complicate the workflow and reduce reproducibility [190]. Furthermore, while MALDI-TOF excels in rapid mass determination and detection of photoproducts or metabolites, it provides limited structural information regarding isomerism, stereochemistry, or conformational changes in photosensitizers. For these aspects, complementary methods such as NMR spectroscopy, high-resolution MS, or X-ray crystallography remain indispensable [78,191]. Another limitation relates to matrix interference: background peaks generated by the organic matrix or endogenous biomolecules (e.g., lipids and proteins in tissue sections) may obscure the signal of interest, particularly in MALDI imaging applications, thus reducing sensitivity and specificity. Quantitative analysis also remains challenging in MALDI, as differences in co-crystallisation, ion suppression effects and variability in laser intensity can affect signal intensity, necessitating the use of internal standards or integration with LC-MALDI workflows [104,117,118,192]. In imaging mass spectrometry, although MALDI-IMS allows for spatial mapping of photosensitizers and therapeutic agents, the achievable spatial resolution (typically 10–50 µm) may still be insufficient to fully capture tumour heterogeneity, subcellular localization, or distribution within complex tumour microenvironments [170,182]. Additional concerns involve the possibility of photodegradation or unwanted fragmentation of photosensitizers during sample preparation or laser desorption, which can introduce artefacts and complicate interpretation of spectra [193]. Finally, from a translational standpoint, while MALDI-based methods are well established in research laboratories, their integration into routine clinical workflows remains limited. Barriers include high instrumentation costs, the need for specialised infrastructure and expertise and the complexity of data processing and interpretation [194]. Overcoming these limitations through technological advances, improved standardisation and multimodal integration will be critical to fully realise the translational potential of MALDI-TOF MS in PDT.

Complementary techniques such as electrospray ionisation mass spectrometry (ESI-MS) and liquid chromatography tandem mass spectrometry (LC-MS/MS) play a critical role. Unlike MALDI, ESI-MS is matrix-free, reduces the risk of ion suppression effects and allows continuous ion generation, making it more suitable for the quantitative analysis of photosensitizers in complex biological matrices [195]. Similarly, LC-MS/MS provides both chromatographic separation and high sensitivity, enabling the detection of low-abundance metabolites and degradation products, as well as accurate quantification [196,197]. The integration of MALDI-TOF MS with these complementary methods can therefore overcome many of its intrinsic limitations, offering a more complete picture of photosensitizer behaviour in preclinical and clinical settings.

In addition to these analytical limitations, practical barriers must be considered for clinical implementation of MALDI-based techniques. Standardisation of sample preparation, matrix selection, instrument settings and data analysis remains a major challenge [198]. The relatively low throughput of MALDI-IMS and multimodal approaches limits large-scale clinical studies [199]. Regulatory validation and formal guidelines for clinical use are still lacking, and the high cost and specialised infrastructure required pose additional hurdles [200]. Finally, the complexity of data processing and interpretation further constrains the translation of these methods into routine clinical workflows [201]. Addressing these challenges will be essential to realise the full potential of MALDI-TOF MS and MALDI-IMS in the clinical context of photodynamic therapy.

A significant limitation that emerged during this review is the relative imbalance in the available literature: the majority of studies applying MALDI-TOF MS to photosensitizers focus primarily on chemical synthesis and structural confirmation, while biological validation is often restricted to basic in vitro assays. Translational investigations, including in vivo studies and clinical applications, remain relatively scarce. This trend is also visible in recent publications, where MALDI is mainly employed for confirming the molecular identity of newly synthesised conjugates, with limited extension toward comprehensive biological or clinical validation [202]. As a consequence, although MALDI-TOF MS demonstrates great promise as an analytical platform for PDT, its clinical relevance is still more prospective than established. Addressing this gap will require systematic studies that integrate MALDI-based analyses with robust in vivo models and clinical trials to fully realise its translational potential.

## 8. Conclusions

The integration of MALDI-TOF mass spectrometry with photodynamic therapy (PDT) opens up new possibilities in the areas of diagnosis, monitoring and optimisation of cancer and infection treatment. MALDI-TOF is applicable at every stage of the development of photosensitisers—from the synthesis and characterisation of new compounds, to the study of their stability and metabolism in vivo, to the analysis of the molecular mechanisms of action of PDT. This technique allows not only the precise verification of the structure and purity of photosensitisers, but also the tracking of changes in the proteome and metabolome of treated cells and the control of next-generation bioconjugates and nanocarriers.

The use of MALDI-IMS additionally allows spatial imaging of the distribution of photosensitisers and drugs in tissues, which is crucial for assessing the bioavailability and efficacy of therapies. MALDI-TOF also provides a valuable tool in microbiological diagnostics, enabling rapid detection of pathogens and supporting the use of PDT in the treatment of infections.

MALDI-TOF MS acts as a versatile analytical platform for PDT because it provides information at multiple stages of the translational pipeline. In the discovery phase, MALDI-TOF enables rapid confirmation of photosensitizer structure, purity and conjugation to carriers, ensuring robust quality control. In preclinical research, MALDI-TOF facilitates monitoring of metabolic stability and photoproducts, while MALDI imaging mass spectrometry (MALDI-IMS) allows spatial mapping of photosensitizer distribution within tumour and healthy tissues, a key determinant of therapeutic efficacy and safety. In the clinical context, MALDI-TOF is already established as a routine diagnostic tool in microbiology laboratories, where it accelerates pathogen identification and supports antimicrobial PDT applications. This continuity from bench to bedside illustrates its direct translational impact: MALDI-TOF not only accelerates the development and validation of novel photosensitizers but also provides clinically relevant data that inform patient selection, dosing strategies and treatment monitoring. Its integration into PDT research highlights how a single analytical platform can bridge basic, preclinical and clinical applications, thus enhancing the personalization and effectiveness of photodynamic therapy.

Looking to the future, the combination of MALDI-MS with other analytical techniques in a multi-omics approach and the development of multimodal molecular imaging may contribute significantly to personalised therapeutic strategies. The results of the studies to date indicate that MALDI not only supports the development of new photosensitisers but also lays the foundation for further refinement and personalisation of PDT, enhancing its efficacy and clinical safety.

## Figures and Tables

**Table 1:** Overview of selected photosensitizers (PS) analysed by MALDI mass spectrometry, including their class, theoretical/provided mass, applied MALDI matrix and the main outcomes of the analyses with relevance to photodynamic therapy (PDT).

Name of PS	Theoretical Mass/Proven Mass	MALDI Matrix	Type of Biological Validation	Main Conclusion of the Analysis/Significance of Potential Use	Source
ANTS-ZnPcp (peripheral Zn-phthalocyanine with Schiff base substituents)	Calc.:1799.23/Found: 1800.24 [M+H]+	-	Not reported	MALDI-TOF confirmed synthesis and structure; PS with high singlet oxygen yield (ΦΔ = 0.53), potential candidate for PDT	[133]
ANTS-ZnPcnp (non-peripheral Zn-phthalocyanine with Schiff base substituents)	Calc.: 1799.23/Found:1800.16 [M+H]+	-	Not reported	MALDI-TOF confirmed synthesis and structure; PS with moderate singlet oxygen yield (ΦΔ = 0.27), potential candidate for PDT	
Zn(II)-phthalocyanine (4,6) with coumarin substituents	Found: ~1434 *m*/*z* [M]+	-	Not reported	MALDI-TOF confirmed structure; compounds well soluble, generate singlet oxygen at acceptable levels, potential PS for PDT	[134]
In(III) acetate-phthalocyanine (5,7) with coumarin substituents	Found: ~1543 *m*/*z* [M]+	-.	Not reported	MALDI-TOF confirmed structure; show good photophysical/photochemical properties, potential PS for PDT	
Bis(4-(6—bromo-2-naphtoxy)-phthalocyaninato- silicon (IV) (SiPc-BrNph)	Calc.: 984.74 g/mol/Found: *m*/*z* 984.52 [M]+	-	Not reported	Novel diaxially substituted SiPc complex with high singlet oxygen generation efficiency (ΦΔ = 0.78 in DMSO, 0.69 in DMF in PDT; ΦΔ = 0.94 in DMSO, 0.81 in DMF in SPDT). Candidate for photosensitiser/photosensitiser in PDT, SDT and SPDT, especially for cancer treatment.	[136]
Ru-Mal-CTX (Ru-NH_2_ conjugate with cetuximab via maleimide)	Found: *m*/*z* 157,661 (conjugate mass); the difference corresponds to ~3 units of Ru per 1 CTX	Sinapic Acid (SA)	In vitro	First described Ru-CTX conjugate in the literature; successful coupling of approximately 3 Ru fragments per 1 Ab. Less photoactive than free Ru-NH_2_, but allows targeted delivery of PS.	[155]
Ru-BAA-CTX (Ru-NH_2_ conjugate with cetuximab via benzoylacrylic acid)	Found: *m*/*z* 158,503 (conjugate mass); the difference corresponds to ~4 units of Ru per 1 CTX	Sinapic Acid (SA)	In vitro	More stable conjugation method; conjugation of approximately 4 Ru fragments per 1 Ab. Similarly to Ru-Mal-CTX, less photoactive than free Ru-NH_2_, but important as a selective PS delivery system.	
Ir-BODIPY	Calc.: *m*/*z* 902.84/Found: *m*/*z* 902.866 [M–PF_6_^−^]	-.	Not reported	MALDI-TOF confirmed the structure of the complexes. The complexes show high singlet oxygen generation efficiency (^1^O_2_) and stronger photosensitising properties than typical BODIPY-PS. The authors indicate potential applications in photodynamic therapy (PDT) and other areas requiring efficient PS.	[138]
Ru-BODIPY	Calc.: *m*/*z* 815.69/Found: *m*/*z* 815.091 [M–2PF_6_^−^]	-.	Not reported		
N-allyl bromoporphyrin (non-metalated)	Calc.: 1414.1415/Found: 1419.8168 [M^+^]	Dithranol or without matrix	In vitro	Highest photocytotoxicity and radiosensitization in the comparisons studied; potential PS for PDT and radiation therapy	[151]
5,10,15,20-Tetrakis(benzo[b]thiophene) porphyrin (BTP)	Calc.: 839.08/Found: 839.2 [M^+^]	-	In vitro	Confirmed formation of target PS in high purity; effective in PDT against MCF-7 cells, generates ROS, induces DNA fragmentation, controlled subcellular localization affects cell death mechanism	[139]
Zn(II) phthalocyanine derivative (Pc1)	Calc.: 1667.10/Found: 1666.11 [M-H]+	-	In vitro	Structure and formation of target PS confirmed; shows phototoxicity to breast cancer cells, potential PS for PDT	[140]
Zn(II) phthalocyanine derivative (Pc2)	Calc.: 1667.10/Found: 1664.64 [M-3H]+	-.	In vitro	Structure and formation of target PS confirmed; shows phototoxicity to breast cancer cells, potential PS for PDT	
Si(IV) phthalocyanine derivative (Pc3)	Calc.: 1311.64/Found: 1339.96 [M+Na+5H]+	-	In vitro	The structure and formation of the target PS was confirmed; intense NMR signals confirm the presence of a Pc ring and chalcone groups, a potential PS for PDT.	
1(4),8(11),15(18),22(25)-Tetrakis(3-(4-methoxy-phenoxy)phthalocyaninato)gallium(III) chloride	Calc.: 1106.18/Found: 1071.68 [M-Cl+H]+	2,5-dihydroxybenzoic acid	In vitro	Structure of target PS confirmed; high solubility in many solvents; potential PS for PDT (singlet oxygen ΦΔ = 0.64)	[156]
1(4),8(11),15(18),22(25)-Tetrakis(3-(4-(methylthio)phenoxy)phthalocyaninato)gallium(III) chloride	Calc.: 1170.45/Found: 1135.88 [M-Cl+H]+	2,5-dihydroxybenzoic acid	In vitro	Structure of target PS confirmed; high solubility in many solvents; potential PS for PDT (singlet oxygen ΦΔ = 0.56)	
Folic acid/hexane-1,6-diamine/4-carboxyphenylporphyrin	Calc.: 1180.494/Found: 1180.47	α-cyano-4-hydroxy-trans-cinnamic acid (CHCA)	In vitro	Conjugate structure confirmed; high selectivity to cells overexpressing the folate receptor; effective PS in PDT (LD50 = 22.6 J/cm^2^)	[157]
Folic acid/2,2′-(ethylenedioxy)-bis-ethylamine/4-carboxyphenylporphyrin	Calc.: 1212.484/Found: 1212.49	α-cyano-4-hydroxy-trans-cinnamic acid (CHCA)	In vitro	Confirmed conjugate structure; increased solubility and biocompatibility; high cellular uptake and effective PS in PDT (LD50 = 6.7 J/cm^2^)	
bis{4-[2-(2H-1,2,3-benzotriazol-2-yl)-4-(1,1,3,3-tetramethylbutyl)phenoxy]} phthalocyaninato silicon(IV) (SiPc)	Calc.: 1185.49 g/mol/Found: 1185.66 *m*/*z*	-_	Not reported	Potential sono-photosensitizer in SPDTs; high singlet oxygen generation quantum yields (ΦΔ) in different solvents; correct molecular structure confirmed	[142]
2(3),9(10),16(17),23(24)-Tetrakis-4-(3,4-dimethoxyphenethyl)-5-ethyl-2H-1,2,4-triazol-3(4H)-one phthalocyaninato zinc(II) (p-ZnPc)	Calc.: 1679.13 g/mol/Found: 1679.44 *m*/*z*	-	In vitro	All PS have a similar structural formula, differing in central metal (Zn, Mg, Pb) and location of substituents (peripheral ‘p’ or non-peripheral ‘np’). Structure confirmed; well soluble; potential PS in PDT	[158]
1(4),8(11),15(18),22(25)-Tetrakis-4-(3,4-dimethoxyphenethyl)-5-ethyl-2H-1,2,4-triazol-3(4H)-one phthalocyaninato zinc(II) (np-ZnPc)	Calc.: 1679.13 g/mol/Found: 1679.21 *m*/*z*	-.	In vitro		
2(3),9(10),16(17),23(24)-Tetrakis-4-(3,4-dimethoxyphenethyl)-5-ethyl-2H-1,2,4-triazol-3(4H)-one phthalocyaninato magnesium(II) (p-MgPc)	Calc.: 1638.06 g/mol/Found: 1638.39 *m*/*z*	-	In vitro		
1(4),8(11),15(18),22(25)-Tetrakis-4-(3,4-dimethoxyphenethyl)-5-ethyl-2H-1,2,4-triazol-3(4H)-one phthalocyaninato magnesium(II) (np-MgPc)	Calc.: 1638.06 g/mol/Found: 1638.54 *m*/*z*	-	In vitro		
2(3),9(10),16(17),23(24)-Tetrakis-4-(3,4-dimethoxyphenethyl)-5-ethyl-2H-1,2,4-triazol-3(4H)-one phthalocyaninato lead(II) (p-PbPc)	Calc.: 1820.95 g/mol/Found: 1820.11 *m*/*z*	-.	In vitro		
1(4),8(11),15(18),22(25)-Tetrakis-4-(3,4-dimethoxyphenethyl)-5-ethyl-2H-1,2,4-triazol-3(4H)-one phthalocyaninato lead(II) (np-PbPc)	Calc.: 1820.95 g/mol/Found: 1820.19 *m*/*z*	-	In vitro		

## Data Availability

No new data were created or analyzed in this study. Data sharing is not applicable to this article.
